# In vitro comparison of antimicrobial activity of aqueous decoction of *Coriandrum sativum*, and Dentol Drop with chlorhexidine on *Streptococcus mutans*

**Published:** 2013-09

**Authors:** Hamid Moradian, Abdollah Bazargani, Azade Rafiee, Ali Nazarialam

**Affiliations:** 1 Department of Pediatric Dentistry, Dental School, Shiraz University of Medical Sciences, Shiraz, Iran; 2 Department of Bacteriology and Virology, Shiraz Medical School, Shiraz University of Medical Sciences, Shiraz, Iran; 3 Student Research Committee, Dental School, Shiraz University of Medical Sciences, Shiraz, Iran

**Keywords:** Antimicrobial Activity, *Streptococcus mutans*, *Coriandrum sativum*, Dentol Drop, chlorhexidine

## Abstract

**Background and objectives:**

Dental caries is still remained as a major health problem. This problem has created a new interest to search for new antimicrobial agents from various sources including medicinal plants. Since limited data is available so far regarding the antibacterial effect of *Coriandrum sativum* seed and Dentol Drop against *Streptococcus mutans*, this study aims to assess this activity.

**Materials and Methods:**

This experimental study was conducted in Shiraz University of Medical Sciences. In vitro comparison of antimicrobial activity of aqueous decoction of *Coriandrum sativum* seed and Dentol drop with chlorhexidine against *Streptococcus mutans* was evaluated using disk diffusion and broth microdilution assays. Positive and negative controls were considered. The data was statistically analyzed by applying Kruskal-Wallis and Tukey post-hoc test to compare the groups using SPSS software (version 17).

**Results:**

Dentol drop showed a remarkable antibacterial activity, in comparison with chlorhexidine, against *S. mutans* in the disk diffusion (p value = 0.005), and broth microdilution assays (p value = 0.0001). Based on the results of this study, *Coriandrum sativum* seed did not posses any antibacterial property.

**Conclusion:**

*Coriandrum sativum* seed showed no anti-*Streptococcus mutans* activity. Dentol drop exhibited a remarkable antibacterial activity against *S. mutans* when tested *in vitro*. Dentol drop can be further studied as a preventive measure for dental caries.

## INTRODUCTION

Dental caries and periodontal problems are the most common chronic diseases worldwide. Dental caries is defined as an infectious bacterial disease that results in destruction of the calcified tissues of the teeth. One of the bacteria attributed to dental caries is *Streptococcus mutans* with eight serotypes. All *S. mutans* serotypes are acidogenic and aciduric and are strongly stimulated by sucrose. It seems that S. mutans is one of the primary organisms associated with dental caries in human. A caries prevention method is a complex process comprised of multiple aspects. Its primary aim is to reduce the numbers of cariogenic bacteria. To reach this goal, limiting substrate, disrupting of plaque formation with brushing and flossing, modifying tooth surface with different forms of fluoride, stimulating the saliva flow, restoring cavitated tooth surface and modifying cariogenic microflora to non-cariogenic ones with topical fluoride treatment, antibiotic treatment or bactericidal mouth rinses such as chlorhexidine can be applied (1). The golden standard for the mouth rinses is a diguanidohexane with pronounced antiseptic properties, named chlorhexidine. Reversible side effects of chlorhexidine use are brown staining of the teeth, transient impairment of taste perception, sore and dry mouth, sloughing of gingival tissue and systemic side effects if swallowed (2). It is also claimed that there is a specific re-colonization pattern of *S. mutans* after chlorhexidine treatment. The re-emergence of *S. mutans* is most probably because of the regrowth of bacteria which have not been eradicated (3).

Recently there has been a renewed interest in the use of herbal mouth rinses (4). One of these herbs is coriander (*Coriandrum sativum*) which traditional medicine has attributed many properties to it including antimicrobial effects (5). In a survey, *Coriandrum sativum* inhibitory effect on *Klebsiella pneumonia* 13883, *Bacillus megaterium* NRS, *Pseudomonas aeroginosa ATCC* 27859, *Staphylococcus aureus* 6538, *Escherichia coli* ATCC 8739, *Enterobacter cloaca* ATCC 13047, *Corynebacterium xerosis* UC 9165, *Enterococcus faecalis* DC 74, *Kluyveromyces marxianus, Rhodotorula rubra* was reported (6, 7). Other studies also considered antimicrobial properties for *Coriandrum sativum* (8, 9). Another study reported that volatile compounds of Coriandrum sativum posses bactericidal activity against *Salmonella cholerae suis* (10). Other studies reported no inhibition zone for the tested microorganisms (11, 12). Another study also showed no antimicrobial effect of *Coriandrum sativum* on oral bacterial isolates (13).

Dentol drop, mainly known for its potential to alleviate dental pain, is derived from *Satureja khusistanica*. This herbal drop is consisted of 10% carvacrol according to the product leaflet. In a study, *Satureja bachtiarica* was reported to posses inhibitory effect to the tested microorganisms (14). Another survey, also reported antimicrobial activity of *Satureja khusistanica* against *Candida albicans* and the tested Gram positive and negative bacteria (15). In the leaflet of Dentol drop a wide spectrum antimicrobial activity against oral pathogens is mentioned but the species names of these organisms are not precisely clarified.

The aim of this study was to investigate the antimicrobial activity of *Coriandrum sativum* seed, and Dentol drop on *S. mutans* in comparison with chlorhexidine to produce an effective, cheap and accessible mouth rinse against *S. mutans* with fewer side effects.

## MATERIALS AND METHODS

This experimental study was conducted in Shiraz University of Medical Sciences. Fresh seeds of *Coriandrum sativum* were purchased from a standard spice supplying herbal company. Dentol drop (Khoraman laboratory, Iran) and chlorhexidine (Behsa, Iran) 0.2% mouth rinse were bought from a local pharmacy. Lyophilized *Streptococcus mutans* ATCC 35668 (PTCC 1683) was purchased from the Persian Type Culture Collection (PTCC), Iran.

### Preparation of aqueous decoction of *Coriandrum sativum seed*

Aqueous decoction of *Coriandrum sativum* seed was prepared by boiling 10 gram of *Coriandrum sativum* seed in 4 L sterile distilled water over low flame until it got to 1-liter volume (17). The flask was then plugged and was removed from heat and was allowed to cool. Then the content of the flask was filtered (16).

### Inoculation of culture media with reference strain

The pure culture of *S. mutans* was prepared in nutrient broth. Lyophilized *S. mutans* was added to Triptycase Soy Broth (TSB) media and was incubated in an appropriate atmosphere (H_2_:CO_2_:N_2_ 10:10:80) at 37°C for 24 hours. Cultivated Bacteria were then streaked onto the Blood agar media. The media were incubated for 24 hours. One well-isolated colony of this reference strain was selected from the agar plate and was aseptically transferred into the 4ml of sterile nutrient broth medium. The broth medium was then incubated in an appropriate atmosphere (H_2_:CO_2_:N_2_ 10:10:80) at 37°C for 24 hours. For antimicrobial susceptibility testing, the turbidity of bacterial suspension must be adjusted equivalent to a 0.5 McFarland standard. A 0.5 McFarland standard is comparable to a bacterial suspension of 1.5×10^8^ CFU/ml.

### Broth microdilution assay

For broth microdilution, susceptibility panel in 96-well µl plates were prepared by dispensing 200 µl of each medicinal herb used in the study with the highest concentrations into the first column wells and 100 µl of nutrient broth into the remaining wells. Then, the two-fold serial dilutions of each medicinal herb solution were made by drawing up 100 µl of each medicinal herb solution in the first column wells into the second column and then move on to the next column to achieve the final concentrations. Then the 0.5 McFarland bacterial suspensions (1 x 10^8^ CFU/mL) were diluted 1:10 to yield 10^7^ CFU/mL. 5 µl of this suspension was inoculated into each well of microtiter plates to obtain the final concentration of bacteria approximately 5 x 10^4^ CFU/well (10^5^ CFU/ml). The last two wells were positive and negative controls, respectively. The positive control was inoculated with bacterial suspension only, while the negative control well was left blank without inoculation. The 96-micro well plates were sealed using a perforated plate seal and incubated in an appropriate atmosphere (H_2_:CO_2_:N_2_ 10:10:80) at 37°C for 48 h. The MIC values were recorded as the lowest concentration where no viability is observed in the wells of 96-micro well plates after incubation period. Each test was done in triplicate.

For minimal bactericidal concentration (MBC) determination of the tested agents, 10 µl of the broth medium in each well that had not shown any growth was collected and inoculated on a fresh blood agar medium. Another blood agar medium was also inoculated with *S. mutans* as a control. The plates were incubated at 37°C for 24 h. The concentration at which no growth was detectable was considered as MBC.

### Disk diffusion method

Each sterile paper disk was impregnated with 30 µl of undiluted *Coriandrum sativum* seed, Dentol drop, and chlorhexidine. The disks were allowed to dry. Using a sterile forceps, the disks were placed on the inoculated blood agar medium. 1 paper disk on each plate was soaked in distilled water as a negative control. Then the plates were incubated in an appropriate atmosphere (H_2_:CO_2_:N_2_ 10:10:80) at 37°C for 24 hours and the diameter of the inhibition zone was measured by a ruler. Each test was done in triplicate.

### Statistical analysis

The results were interpreted with the standard deviation. The data was statistically analyzed by employing Kruskal-Wallis and Tukey post-hoc test to know the significant difference in antimicrobial susceptibility of each herbal solution using SPSS software (version 17, Chicago, IL, USA).

## RESULTS

This experimental study was conducted to evaluate the antibacterial activity of *Coriandrum sativum* seed, and Dentol drop, in comparison with chlorhexidine on *S. mutans*. The MIC values of *Coriandrum sativum* seed, Dentol drop and chlorhexidine are presented in Table 1. Based on Tukey post-hoc test (Table 2), Dentol drop showed greater antibacterial effect than *Coriandrum sativum* seed (p value = 0.0001). However; the MIC value of Dentol drop and chlorhexidine did not show a significant difference. This means that antibacterial activity of Dentol drop is somehow as great as chlorhexidine. MIC value of Coriander seed was higher than 0.5 i.e. all concentrations of coriander showed bacterial growth in wells of microtiter plates, and higher concentrations were not assessed in this study. The concentrations used for MIC determination ranged from 0.5-0.0002 µg/ml for *Coriandrum sativum*, 1-0.00048 µg/ml for chlorhexidine, and 5-0.0024 µg/ml for Dentol drop. For MBC determination, the concentration at which no growth was seen on blood agar was the same as MIC value for each tested agents. So we considered the MBC equal to MIC for chlorhexidine and Dentol drop.

**Table 1:** MIC values (µg/ml) of Dentol drop and chlorhexidine determined by broth microdilution assay.

variable	MIC value (µg/ml)	P value
**Dentol drop**	0.0039	0.018
**chlorhexidine**	0.0024	

**Table 2:** Comparison of MIC differences of Dentol drop and chlorhexidine determined by broth microdilution assay.

variable		MIC difference	P value
**Chlorhexidine**	Dentol drop	0.0015	0.949
**Dentol drop**	Chlorhexidine	0.0015	0.949

The results of disk diffusion assay are presented in Table 3. In this method, no inhibition zone was measured for *Coriandrum sativum* seed. The inhibition zone diameter for Dentol drop was larger than chlorhexidine, and coriander seed (p value = 0.005).

**Table 3:** The average and standard deviation of inhibition zone diameters (mm) of *Coriandrum sativum* seed, Dentol drop and chlorhexidine determined by disk diffusion assay.

variable	Inhibition zone diameter (mm)	P value
***Coriandrum sativum***	0	0.005
**chlorhexidine**	9.75 ± 0.5	
**Dentol drop**	15.75 ± 0.5	

## DISCUSSION

*Coriandrum sativum and Satureja khusistanica* has been widely studied for their antimicrobial activity (6–15). However, a limited data is available so far regarding their antibacterial activity against *S. mutans*. In the present study, Dentol drop showed a remarkable antibacterial activity, in comparison with chlorhexidine, against *S. mutans* in the disk diffusion, broth microdilution assays. Based on the results of this study, *Coriandrum sativum* did not show any antibacterial activity against *S. mutans*.

Kubo *et al*. studied anti- *Salmonella cholerae suis* activity of 13 volatile compounds of fresh leaves of *Coriandrum sativum*. 11 of these volatile compounds were straight chain aldehydes and typical products of oxidative cleavage of unsaturated fatty acids. All the aldehydes tested were reported to be effective against *Salmonella cholerae suis* (10). Our study investigated the antibacterial activity of fresh seeds of *Coriandrum sativum* on *S. mutans*. It is reported that coriander seed oil contains linalool (60-70%) and hydrocarbons (20%) and the composition of the leave oil is completely different from the seed oil (17). This may explain why the result of our study differs.

Keskin and Toroglue studied the antimicrobial activity of the ethyl acetate, acetone and methanol extract of 12 plant species against 2 fungi and 8 bacterial species by disk diffusion assay. *Coriandrum sativum* showed 7-8 mm inhibition zone to the organisms tested (6, 7). Lo cantore et al. (8) and Singh et al. (9) also considered antimicrobial properties for *Coriandrum sativum*. Essential oil extracts of seven spices against 8 bacteria were studied by Singh *et al*. and *Coriandrum sativum* showed good antibacterial activity against all the microorganisms except *Corynebacterium diphtheria*. It is claimed that there are differences in antibacterial activity of plant groups due to the phytochemical differences between species and collection sites. Moreover, there are differences in the antimicrobial effects of different plants to different microorganisms due to the cell wall structure, species and subspecies (6).

Sagdic *et al*. tested 18 extracts of plant spices commonly grow in Turkey including *Coriandrum sativum* against 23 microorganisms. *Coriandrum sativum* did not show bactericidal activity (12). Ates *et al*. studied 5 plants extracts against 13 bacteria. *Coriandrum sativum* had no antibacterial effect to the microorganisms tested (11). Chaudhury et al. used aqueous decoction of 4 plants against oral pathogens. Similar to our results, *Coriandrum sativum* did not exhibit any antibacterial activity to the tested organisms (13).

Mohammadpour *et al*. investigated antimicrobial activity of *Satureja bachtiarica* and reported its inhibitory effect on the microorganisms tested (14). Dosty *et al*. investigated antimicrobial activity of *Satureja khusistanica* and found antimicrobial activity against *Candida albicans* and the Gram positive and negative bacteria tested (15). The antimicrobial activity of *Satureja bachtiarica* and *Satureja khusistanica* is mainly attributed to carvacrol and thymol which are phenolic monoterpenes (14). According to the product leaflet, Dentol drop contains 10% carvacrol and is derived from *Satureja khusistanica*.

In many previous studies, only disk diffusion method was used; however *Coriandrum sativum seed* and Dentol drop were reported to be effective against different microorganisms without mentioning the MIC values (6–15). Besides, it has been reported that the disk diffusion method is not always reliable for determining the antimicrobial property of natural antimicrobials because the polarity of natural compounds can affect the diffusion of compounds on the culture medium. Less polar compounds diffuse slower than more polar ones (18) Therefore; we used two different methods in our study. The differences in the temperature while preparing agar plates may also affect the results. The constituents of the seeds may vary as the age increases.

According to the results of this study, Dentol drop has a remarkable inhibitory effect on *S. mutans* growth. However; toxicity, bioavailability and in vivo efficacy of Dentol drop must be evaluated before using it as a preventive measure for dental caries.

In Conclusion, *Coriandrum sativum* seed showed no anti-*Streptococcus mutans* activity. Dentol drop exhibited a remarkable antibacterial activity against *S. mutans* when tested *in vitro*. Dentol drop can be further studied as a preventive measure for dental caries.
